# Medical Abortion Provided by Nurse-Midwives or Physicians in a High Resource Setting: A Cost-Effectiveness Analysis

**DOI:** 10.1371/journal.pone.0158645

**Published:** 2016-06-30

**Authors:** Susanne Sjöström, Helena Kopp Kallner, Emilia Simeonova, Andreas Madestam, Kristina Gemzell-Danielsson

**Affiliations:** 1 Division of Obstetrics and Gynecology, Department of Women’s and Children’s Health, Karolinska Institutet, Karolinska University Hospital, Stockholm, Sweden; 2 Department of Obstetrics and Gynecology, Department of Clinical Sciences at Danderyds Hospital, Karolinska Institutet, Stockholm, Sweden; 3 John Hopkins University, Carey School of Business, Baltimore, Maryland, United States of America; 4 Stockholm University, Department of Economics, Stockholm Sweden; NHS Lothian and University of Edinburgh, UNITED KINGDOM

## Abstract

**Objective:**

The objective of the present study is to calculate the cost-effectiveness of early medical abortion performed by nurse-midwifes in comparison to physicians in a high resource setting where ultrasound dating is part of the protocol. Non-physician health care professionals have previously been shown to provide medical abortion as effectively and safely as physicians, but the cost-effectiveness of such task shifting remains to be established.

**Study design:**

A cost effectiveness analysis was conducted based on data from a previously published randomized-controlled equivalence study including 1180 healthy women randomized to the standard procedure, early medical abortion provided by physicians, or the intervention, provision by nurse-midwifes. A 1.6% risk difference for efficacy defined as complete abortion without surgical interventions in favor of midwife provision was established which means that for every 100 procedures, the intervention treatment resulted in 1.6 fewer incomplete abortions needing surgical intervention than the standard treatment. The average direct and indirect costs and the incremental cost-effectiveness ratio (ICER) were calculated. The study was conducted at a university hospital in Stockholm, Sweden.

**Results:**

The average direct costs per procedure were EUR 45 for the intervention compared to EUR 58.3 for the standard procedure. Both the cost and the efficacy of the intervention were superior to the standard treatment resulting in a negative ICER at EUR -831 based on direct costs and EUR -1769 considering total costs per surgical intervention avoided.

**Conclusion:**

Early medical abortion provided by nurse-midwives is more cost-effective than provision by physicians. This evidence provides clinicians and decision makers with an important tool that may influence policy and clinical practice and eventually increase numbers of abortion providers and reduce one barrier to women’s access to safe abortion.

## Introduction

Lack of trained providers is a common barrier to safe provision of abortion. Shortage of physicians as well as physicians’ unwillingness to provide abortion is common in rural areas, but is also becoming an increasing problem in high resource environments and in settings where abortion is legal [1] [2]. Unsafe abortion is estimated to cause eight percent of global maternal mortality, implying that 23000 preventable deaths occur each year [3]. Thus eliminating barriers to women’s access to safe abortion is an important tool to reduce maternal mortality. The number of providers of safe abortion can be increased, by task-shifting abortion provision from physicians to health care providers with adequate but less training [4, 5]. Non-physician providers have been shown to provide medical abortion as effectively and safely as physicians in different settings[6–8]. Medical abortion using mifepristone and misoprostol is highly effective, and is recommended by the World Health Organization (WHO) [9].

Health care budgets are limited worldwide and determining the cost effectiveness of an intervention is, together with aspects such as efficacy, safety and accessibility, important in order to influence policy and clinical practice. One previous study from a high resource setting using a mifepristone-misoprostol regimen found medical abortion to be less costly than surgical methods [10]. The cost-effectiveness of medical abortion performed by non-physician providers compared to provision by physicians using a mifepristone and misoprostol regimen with WHO recommended dosages, remains to be evaluated.

The objective of our study was to conduct a cost-effectiveness analysis of medical abortion provided by nurse-midwifes or physicians in a high resource setting where ultrasound examination and dating of pregnancy is part of the protocol.

## Material and Methods

### Study background

This study is based on a previously published randomized two-sided equivalence study by Kopp Kallner et al including 1180 healthy women seeking treatment for abortion at an out-patient clinic of a university hospital in Sweden between February 2011 and July 2012 [8]. Eligible participants willing to undergo medical abortion were randomized to the intervention where a nurse-midwife counseled, examined, informed, and treated the woman (n = 597), or the standard treatment, in which counselling and physical examination was provided by a physician, and a nurse-midwife gave additional information and medication (n = 583). Contraceptive counselling and prescription was provided by the allocated care-giver. All women were treated with mifepristone 200mg orally at the clinic on day one, and misoprostol 800mcg vaginally at home or in the clinic 24–48 hours after the mifepristone administration. Follow up was provided by a nurse-midwife not participating in the study approximately three weeks after the mifepristone administration using a low sensitivity urinary human chorionic gonadotropin (u-hCG) test (cut-off 500 IU/ml). Outcome measures in the parent study were efficacy defined as complete abortion without need for surgical intervention, safety defined as no complication, and acceptability defined as preferred provider, should the woman have an abortion in the future. Complication was defined as the need for causal treatment at an unscheduled visit within 6 weeks after the abortion. Efficacy and safety were assessed using electronic patient records and self-administered questionnaires that were also used for measuring acceptability. The duration of the patients’ initial visit to the clinic, as well as the allocated provider’s need for a second opinion from a doctor, was recorded in the study protocol. Statistical calculations were made using SPSS 20 (IBM Corporation, Somers, NY, USA) except the generalized estimating equation performed with SAS 9.3 (SAS Institute, Gary, NC, USA).

### Details of ethical approval

Permission was granted by the National Board of Health and Welfare and by the Ethical Review Board of Stockholm (permission number 2010/1828-31/3, 23 December 2010) to allow midwifes to independently provide medical TOP, according to the study protocol. After approval by the regional ethics committee at Karolinska Institutet all applications were publicly available. The study was registered with Clinicaltrials.gov NCT01612923.

### Cost-effectiveness analysis

The main goal of the present study is to examine the cost-effectiveness of the standard versus the intervention treatment for medical abortion using direct costs. The secondary aims are to evaluate indirect costs, the costs of the subsequent patient’s waiting time and the cost of complications. Direct costs were calculated for the woman’s first visit to the clinic where she was treated according to the standard or intervention arms. Treatment on day 2 (24–48 hours after mifepristone) and follow-up did not differ between groups. We calculated the incremental cost effectiveness ratio (ICER) to determine the cost for achieving a complete abortion without surgical intervention when adopting the intervention as compared to the standard treatment. The ICER considers changes in effectiveness as well as cost of treatment and was established using the formula: [*Cost of Intervention*–*Cost of Standard treatment*]/ [*Effectiveness Intervention*–*Effectiveness of Standard treatment*] where Intervention is nurse-midwife provision of medical abortion, and the Standard treatment is abortion provision by physicians. The Effectiveness is the difference in numbers of complete abortions without surgical intervention between the groups, which was measured as 1.6 fewer abortions requiring vacuum aspiration for completion per 100 patients in the Intervention group in the parent study. It is important to conduct a cost-effectiveness analysis even though the effectiveness of the Intervention treatment has already been established. The costs of the two treatments could still be significantly different. If the Intervention treatment is substantially more expensive than the Standard treatment, the difference in efficacy might not be enough to justify its wide-spread adoption.

A model taking into account the cost of midwives and physicians (based on salaries, payroll tax, and time spent with the patients), usage of surgery rooms, consultation time for second opinion with a physician or senior physician, cost of the treated women’s time, and training of participating midwives was constructed. Direct costs of salaries and examination room rent were derived from the department of Women’s and Children’s Health, Karolinska University Hospital, Stockholm, Sweden in 2011. The cost of consultations was calculated based on the average consultation time and participating physicians’ average salary. The Statistics Sweden (Statistiska Centralbyrån, SCB) report”Average income among women per age group in Stockholm County 2011” was used to estimate the cost of the treated women’s time. Searches in the SCB database were conducted in February and November 2014 [11]. The costs of training the nurse-midwifes were assessed based on the cost of a subsequently developed ultrasound course for midwifes providing medical abortion, including the salary costs of the participating midwifes and the cost of physician supervision. The cost of complications was calculated based on recorded unscheduled visits using the registered diagnosis and treatment according to the diagnostic-related group-coding system, a weighted average of costs per diagnostic related group (DRG) (www.socialstyrelsen.se). There was no difference in costs of disposables, ultrasound, or medication. The comparison was made per procedure. To derive a conservative estimate of the cost of the intervention the reduced waiting time of the subsequent patient was added.

All costs were calculated in Swedish Krona (SEK) and converted to 2011 Euro (EUR) (average exchange rate 2011; 1 Euro € = SEK 9.0298).

## Results

The randomized-controlled equivalence trial showed that provision of medical abortion by nurse-midwifes was superior to provision by physicians, with a risk difference for effectiveness, complete abortion without surgical intervention, of 1.6% (95% CI; 0.2–3.6%, *p* = 0.027). This means that for every 100 patients (procedures), the Intervention treatment resulted in 1.6 fewer follow-up surgical abortions than the Standard treatment. In per-procedure terms, this translates into 0.016 fewer surgical interventions per treatment in the Intervention arm. The woman’s initial visit to the clinic was significantly less time consuming in the intervention arm (average 42 minutes) than the standard treatment (60 minutes) (P<0.001), which affects most direct cost parameters. Patients allocated to the standard treatment spent, on average, the same amount of time (30 minutes) with each provider. The direct costs of the woman’s first visit to the clinic of the standard treatment was EUR 58.3 per procedure and the cost of the intervention treatment was EUR 45, see table 1.

**Table 1:** Direct costs of standard and intervention treatment, per procedure, 2011 EUR.

Cost item	Cost Standard n = 533	Cost Intervention n = 535
**Salary midwife**	**18**	**25**
**Salary physician**	**21**	**0**
**Examination room**	**5**	**4**
**Consultation w physicians**	**0.3**	**1**
**Patient’s time**	**14**	**10**
**Training of midwives**	**0**	**5**
**Total cost per procedure EUR**	**58.3**	**45**

The direct costs are dependent on treatment time mean 60 minutes (SD18.3) in the standard care group, and 42 minutes (SD 24.1) in the intervention group(P<0.001)

Training costs are based on the total cost of training for the 2 participating midwives and include the cost of a 2-day course, salary and physician supervision of 50 ultrasounds. Total EUR 2774, 535 procedures were performed during study

Consultations, when the caregiver obtained a second opinion from another physician, occurred in 4% of the cases for doctors and in 26% for nurse-midwifes (95%CI 18–26%, P<0.001). The physician providers’ consultations lasted 14 minutes on average, 17% of the consultations were ultrasound queries. Less experienced physicians consulted more often than more senior physicians. Nurse- midwifes consultation lasted six minutes on average and 42% the consultations were ultrasound queries, which decreased over time. Costs of the time women spent at the clinic were assessed using the time women spent with their allocated provider and the average income among women per age group in Stockholm County, adjusted to participating women per age group. Women’s travel time was not included. The nurse-midwives participating in the study were highly experienced in all aspects of abortion care, including contraceptive counselling and insertion of IUDs and implants, but had not previously performed ultrasounds. In the study setting, participating nurse-midwives were introduced to the concepts of ultrasound by a senior consultant, and coupled with physicians performing ultrasounds. No additional cost was associated with training of the physicians.

In the Intervention arm the waiting time for future abortion seeking women was reduced by 0.3 hours, which further reduces the cost of the intervention by EUR 4. This is assuming that these women have the same age distribution and the same average income as the women included in the study.

The overall complication rate, defined as an unscheduled visit for symptoms that lead to further treatment was 4.1% (20/493) in the nurse-midwife group and 6.1% (29/472) in the standard care group (95%CI; -0.7–5%, *p* = 0.14). There were no significant differences in safety parameters, and costs for complications were thus not included in the ICER calculations. In the intervention group, 13 (2.6%) women were treated as outpatients and 6 (1.2%) admitted to the hospital. In the standard care group, 21 women (4.4%) were treated at the outpatient clinic and 7 patients admitted (1.5%). Secondary costs of unscheduled visits, complications and surgery were derived from the hospital files for each patient. Costs of complications are shown in table 2.

**Table 2:** Cost of complications 2011 EUR.

Treatment	Standard (n = 533)*	Intervention (n = 535)**
**Outpatient**	10122 (n = 21)	5306 (n = 13)
**Clinic**	15362 (n = 7)	14273 (n = 6)
**Total**	25485	19579
**Per procedure**	48	37

* 2 missing, 1 patient had 2 complications. One of the women in the physician group was treated as an outpatient and later admitted to the hospital.

**1 missing A woman in the intervention group sought care outside of Stockholm county thus treatment costs could not be tracked.

The previous study has established that nurse-midwife provision of medical abortion to healthy women in a high resource setting where ultrasound dating of the gestational length is part of the protocol is more efficacious than provision by physicians. Calculation of direct and indirect costs show that the intervention is also cheaper. Based on these findings a negative ICER ranging from -831.25 EUR when only direct costs are considered to -17500 EUR evaluating total costs was calculated. This means that by implementing the intervention a saving in the range of 831 to 1768.75 EUR is obtained for each avoided surgical intervention, see table 3.

**Table 3:** Incremental cost effectiveness ((ICER) of different measures of costs, EUR.

Item	Difference in costs per case	Difference in efficacy per case	ICER
**Direct cost per woman treated**	45–58.3	0.016	-831.2
**Direct costs including waiting time for the consecutive patient**	41–58.3	0.016	-1081.2
**Total direct and indirect costs**	78–106.3	0.016	-1768.8

The parent study found that women in the nurse-midwife group had long-acting reversible contraceptives (LARCs) inserted within 3 weeks of the TOP significantly more often than women counselled by physicians (95% CI 3.2–15.2, P = 0.005). Calculations of direct savings due to this finding have not been performed within the scope of the present study but its implication is further discussed below.

## Discussion

We have shown that provision of medical abortion by nurse-midwives is cost-saving and equally effective as provision by physicians in a high resource setting. These findings are important as the evidence can influence policy-makers’ decisions, change clinical practice in settings where health care budgets are limited, and eventually contribute to increase numbers of abortion providers that are needed in both high and low resource settings. Previous studies have demonstrated that task-shifting of different health care services, such as treatment of orthopedic conditions in low income environment, is cost effective, but this is the first study showing that task-shifting is cost effective in provision of medical abortion [12].

In our study, task shifting of medical abortion generated direct economic benefits associated with the shorter time spent in the clinic by providers and patients and nurse-midwives’ lower salaries. Costs were also reduced due to shorter waiting time for subsequent patients and a possible lower cost for the treatment of complications. The incremental cost effectiveness was negative, which occurs when the intervention is superior in efficacy and cheaper than the standard treatment. In addition, the costs of the intervention are expected to decrease further over time as the cost of training nurse-midwives is disseminated on more procedures and the number of consultations with physicians’ decline further.

Medical abortion has been shown to enhance access to safe abortion and to be cost-effective[13]. It is preferred by women who want to avoid surgery and find the method natural and private. Some previous studies comparing medical and surgical methods of abortion provision and treatment of miscarriage have argued that the cost effectiveness of medical methods is reduced due to complications and method failure[14, 15]. However, complication rates of both medical and surgical termination of pregnancy are low, and dependent on the choice of treatment regimens as well as method of follow-up. Mifepristone-misoprostol regimens are more effective than misoprostol alone and ultrasound can be difficult to interpret leading to unnecessary surgical interventions [16]. Our study using a mifepristone and misoprostol regimen and a 3-week follow up shows an overall efficacy of 98.2%, which is in line with WHO guidelines.

The women in the nurse-midwife group had long-acting reversible contraceptives (LARCs, implants and intrauterine devices and systems) inserted within 3 weeks of the abortion significantly more often than women counseled by physicians (95% CI 3.2–15.2, P = 0.004). It has previously been shown that LARC use decreases numbers of repeat abortions [17]. The type of abortion provider as well as their training in insertion and counseling has previously been shown to be an important factor influencing women’s uptake of LARCs [18, 19] [20]. Approximately 90% of all mortality and morbidity related to abortion could be averted by the use of effective contraception [21]. LARCs are more effective in preventing unintended pregnancies than oral contraceptives and even modest increases in the uptake of LARCs in women of fertile age have been shown to generate cost-savings for societies. A study from the United States shows that if 10% of women aged 20–29 years switched from oral contraception to LARC, total costs for unintended pregnancies would be reduced by USD 288 million per year[22] and a recent study from Norway estimated cost-savings generated from a 5% increased LARC use in women 15–24 years to be 7.2 million NOK or almost 800000 Euro. Rates of unintended pregnancies in the United States have decreased from 51% to 45% between 2008 and 2011, most likely due to increased use of long acting reversible contraception [23]. A recent study showed that a high proportion (24%) of women sought a repeat abortion within four years after a first abortion, fewer repeat abortions were seen among women who started long acting contraception after the index abortion[24]. Repeat abortions and unintended pregnancies that are continued soon after a first abortion, particularly among teenaged and young women, could be significantly reduced by of LARCs

### Strengths and limitations

To our knowledge, this is the first study to evaluate the cost effectiveness of first trimester medical abortion provided by nurse-midwifes or physicians in a high resource setting where ultrasound dating of gestational age is part of the protocol. The study is a straightforward analysis of actual direct costs based on institutional prices derived from the outpatient clinic, where the previous equivalence trial was carried out.

Possible limitations associated with the parent study include that only healthy women were randomized and that the study was not blinded. The participating nurse-midwives were highly experienced and motivated, which might have affected outcomes such as acceptability and higher prescription and provision of LARCs. Neither the parent study, nor the present one provide conclusive answers on why nurse-midwives provide abortion more effectively than physicians. As suggested in the parent study a possible reason women prefer nurse-midwifes may be that abortion is not seen as a medical condition. The shorter time for the first visit in the intervention arm may be associated with women seeing only one provider which reduces waiting times at the medical office. Implementing task-shifting in abortion provision it is important to consider that non-physician providers’ willingness to participate in abortion provision vary between individuals and settings. Two large surveys from California and India respectively indicate that mid-level providers are willing to be trained to perform medical abortions [25, 26], on the other hand a systematic review covering health care providers attitudes toward induced abortion in sub-Saharan Africa and South-East Asia found that nurses and midwives disliked being involved in abortion provision[27]. Savings following implementation of the intervention might be underestimated as we did not consider the opportunity cost due to released physician time. Neither did we assess the gain from increasing the total number of abortions provided. Reduced quality of life and societal costs related to delaying the procedure for those waiting to undergo an abortion are difficult to quantify and beyond the scope the current analysis. As patients are free to seek care at their own discretion it was not possible to trace the costs for all unscheduled visits to clinics outside the Karolinska University Hospital which might have occurred after the follow-up visit.

## Conclusion

Provision of medical abortion by nurse-midwives is more cost effective than the standard treatment, provision by physicians, in a high resource setting where ultrasound dating is part of the protocol. This finding supports previous evidence of the efficacy and acceptability or task shifting in medical abortion, and provides decision-makers and clinicians with an important tool when assuring increased access to safe abortion services.
